# Multiple High-Risk HPV Infections and Opportunistic HPV Prevalence Among Women Aged 18–25 Years with NILM, ASC-US, or LSIL Cytology: A 2024 Cross-Sectional Study

**DOI:** 10.3390/pathogens15090949

**Published:** 2026-09-07

**Authors:** Erik Kudela, Tomas Rokos, Erik Kozubik, Terezia Pribulova, Filip Piovar, Kamil Biringer

**Affiliations:** Clinic of Gynecology and Obstetrics, Jessenius Faculty of Medicine in Martin, Comenius University in Bratislava, 036 01 Martin, Slovakia; erik.kudela@uniba.sk (E.K.); erik.kozubik@uniba.sk (E.K.); pribulova.terezia@gmail.com (T.P.); fil.piovar@gmail.com (F.P.); kamil.biringer@uniba.sk (K.B.)

**Keywords:** human papillomavirus, multiple HPV infections, high-risk HPV, cervical cytology, NILM, ASC-US, LSIL, young women, Slovakia, cobas 4800

## Abstract

Multiple high-risk human papillomavirus (hrHPV) infections in young women are poorly characterized across age and cytology, particularly with partial genotyping. We assessed multiple hrHPV infections among HPV-positive women aged 18–25 years with NILM, ASC-US, or LSIL; the secondary objective was hrHPV positivity among electively tested women with NILM. This retrospective cross-sectional study analyzed 2024 laboratory data from northern Slovakia. Of 7613 unique women, 7574 met cytological eligibility criteria; 804 underwent cobas 4800 testing, and 295 were hrHPV-positive. Multiple infection, defined as concurrent detection of at least two reportable categories (HPV16, HPV18, or other hrHPV), occurred in 69/295 women (23.4%; 95% CI, 18.9–28.5%). No statistically detectable variation was observed by cytology after age adjustment (*p* = 0.471) or by age after cytology adjustment (*p* = 0.891). Among women with multiple infections, HPV16 combined with other hrHPV was the most frequent pattern (30/69; 43.5%). Among 396 electively tested women with NILM, 75 were hrHPV-positive (18.9%; 95% CI, 15.4–23.1%); positivity was lower at ages 23–25 than 18–22 (10.6% vs. 26.6%; OR, 0.33; 95% CI, 0.19–0.57; *p* < 0.001). The NILM estimate describes a self-selected preventive cohort and is not population-representative.

## 1. Introduction

Persistent infection with carcinogenic human papillomavirus (HPV) is the necessary biological precursor of most cervical cancers, whereas newly acquired infections in young women are common and frequently transient [1,2,3]. The transition from cytology-only screening to molecular HPV testing has increased the ability to identify women carrying high-risk HPV (hrHPV), but it has also made the interpretation of concurrent viral types increasingly relevant. Cytology remains a clinically informative correlate: HPV positivity is expected to be higher in atypical squamous cells of undetermined significance (ASC-US) and low-grade squamous intraepithelial lesion (LSIL) than in negative for intraepithelial lesion or malignancy (NILM) specimens [1,4].

Multiple HPV infections are not a uniform exposure. Their observed frequency depends on age, sexual and immunological factors, vaccination, the number of genotypes interrogated, and whether the assay provides full or partial genotyping [5,6,7,8]. Large epidemiological studies have reported substantially different proportions of multiple infections among HPV-positive women, from approximately one fifth to more than two fifths [6,7]. Moreover, evidence does not consistently support biological synergy between concurrent genotypes for high-grade disease; several studies instead suggest that genotype-specific oncogenicity, especially that of HPV16, remains the dominant determinant [8,9,10].

In 2023, a large Northern Slovak analysis documented high hrHPV positivity in young women and described combinations of HPV16, HPV18, and a pooled group of other hrHPV types [1]. However, ages 18–25 encompass a period of marked changes in HPV exposure, immune response, vaccination coverage, and screening indications. Organized cervical cancer screening in Slovakia targets women aged 23–64 years [1]. Age 18 was the lower boundary of the available adult laboratory cohort. Including women aged 18–22 allowed us to describe routine clinical testing before organized screening eligibility. Their inclusion should not be interpreted as support for routine HPV screening below age 23. Single-year estimates may clarify whether multiple infections change meaningfully within this narrow interval.

The primary objective was to estimate the proportion and reportable-category patterns of multiple hrHPV infections among HPV-positive women aged 18–25 years with NILM, ASC-US, or LSIL cytology. Secondary objectives were to estimate hrHPV positivity among cytologically negative women who elected testing during preventive examinations, describe HPV positivity in ASC-US and LSIL, and provide a balanced age-group summary using the observed median age. The categorical single-year analysis was the primary age analysis, while the median-based comparison was secondary. Because the analysis was cross-sectional and partially genotyped, it was not designed to estimate persistence or progression risk.

## 2. Materials and Methods

### 2.1. Study Design, Setting, and Reporting

This retrospective cross-sectional study analyzed cervical cytology and hrHPV DNA results from 7613 unique women aged 18–25 years examined between 1 January and 31 December 2024 in the Žilina region of northern Slovakia. All samples were processed at Martinské Bioptické Centrum, s.r.o. (Zilina, Slovakia). The analytical dataset was supplied as aggregate counts stratified by single-year age and cytological category. The study used the same sampling, cytological classification, and HPV-testing methods as the 2023 regional cohort reported by Kudela et al. [1] and is reported in accordance with the principles of the Strengthening the Reporting of Observational Studies in Epidemiology (STROBE) statement [11].

### 2.2. Study Population

**Figure 1:**
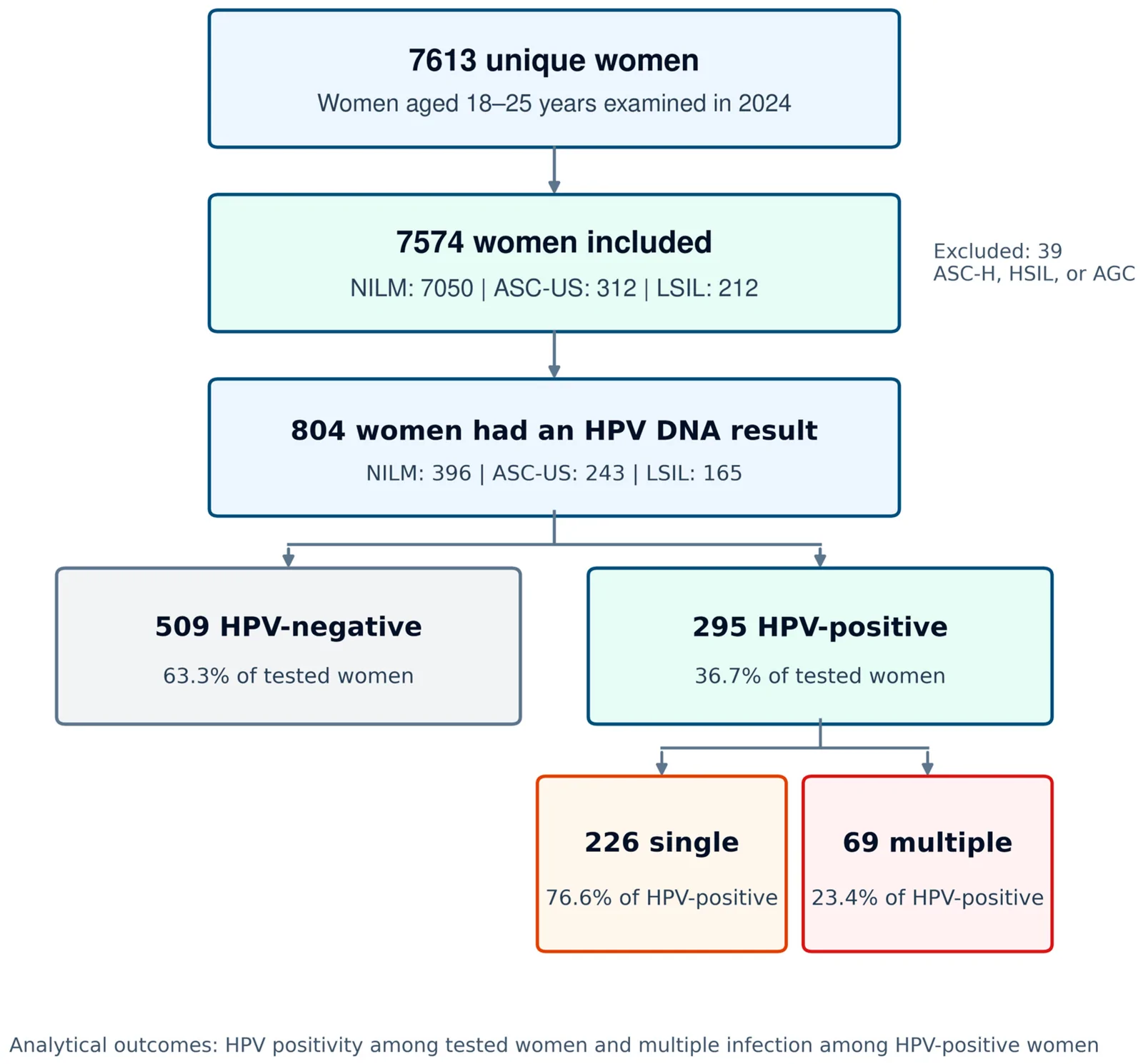
Study flow and analytical denominators. Opportunistic prevalence was estimated in electively tested NILM preventive attendees; overall HPV positivity was described among all tested women, and multiple infection among HPV-positive women. ASC-H, atypical squamous cells—cannot exclude HSIL; ASC-US, atypical squamous cells of undetermined significance; AGC, atypical glandular cells; HPV, human papillomavirus; HSIL, high-grade squamous intraepithelial lesion; LSIL, low-grade squamous intraepithelial lesion; NILM, negative for intraepithelial lesion or malignancy.

The source dataset comprised 7613 unique women aged 18–25 years. Age 18 represented the lower boundary of adult records available in the laboratory dataset, while age 25 defined the upper boundary of the young-women research question. The prespecified analytical population was restricted to NILM, ASC-US, and LSIL cytology, yielding 7574 women: 7050 with NILM, 312 with ASC-US, and 212 with LSIL. Thirty-nine women with ASC-H, HSIL, or AGC were excluded because the research question focused on negative, equivocal, and low-grade cytology. Women aged 18–22 were included to describe routine testing outside organized screening eligibility, not to evaluate or recommend screening below age 23. Among women with NILM, HPV testing was performed at the woman’s request during a preventive gynecological examination rather than because of cytological abnormality. The overall HPV-positivity analysis included 804 women with a definitive HPV result, and the multiple-infection analysis included 295 HPV-positive women (Figure 1). Each woman contributed one record. Vaccination, smoking, sexual behavior, immunosuppression, and follow-up histology were unavailable.

### 2.3. Cytology and HPV DNA Testing

As in Kudela et al. [1], cervical specimens collected during gynecological examinations were processed using either liquid-based cytology (LBC), with specimens placed in a ThinPrep^®^ Pap Test vial containing PreservCyt^®^ Solution (Hologic, Marlborough, MA, USA), or conventional cytology, with specimens fixed directly onto glass slides. Both methods were represented, but the aggregate source dataset did not identify the preparation method for individual women; method-specific counts and analyses were therefore unavailable. Cytological diagnoses were extracted from final routine reports issued by the single diagnostic laboratory and classified according to the 2014 Bethesda System [4]. The aggregate research dataset contained no reader identifiers or records permitting a study-specific blinded re-review, duplicate interpretation, consensus adjudication, or assessment of interobserver agreement.

All HPV DNA analyses were performed using the cobas^®^ HPV DNA test on the cobas^®^ 4800 System (Roche Molecular Systems, Inc., Branchburg, NJ, USA). Cervical cell samples were stored in Roche Cell Collection Medium (Roche Molecular Systems, Inc., Branchburg, NJ, USA) or PreservCyt^®^ Solution (Hologic, Marlborough, MA, USA). This automated qualitative PCR and nucleic-acid hybridization assay detects 14 high-risk HPV types and provides three separate reportable results: HPV16, HPV18, and a pooled result for HPV31, HPV33, HPV35, HPV39, HPV45, HPV51, HPV52, HPV56, HPV58, HPV59, HPV66, and HPV68 [1,5,12]. A positive result was defined as positivity in any reportable category.

### 2.4. Outcome Definitions

The primary outcome was multiple hrHPV infection among HPV-positive women. A single-category result was defined as positivity in only one reportable category: HPV16 only, HPV18 only, or pooled other hrHPV only. A multiple infection was operationally defined as positivity in at least two reportable categories: HPV16 + HPV18, HPV16 + other hrHPV, HPV18 + other hrHPV, or HPV16 + HPV18 + other hrHPV. Because the pooled ‘other hrHPV’ category can contain one or more of 12 genotypes, an ‘other hrHPV only’ result may conceal multiple genotypes. The study therefore cannot determine the true number of individual HPV genotypes present.

The secondary prevalence outcome was hrHPV positivity among electively tested NILM women from preventive examinations. This was interpreted as opportunistic prevalence among cytologically negative preventive attendees. HPV positivity in ASC-US and LSIL and the combined cohort of 804 tested women was treated as test yield rather than population prevalence because the indication and probability of testing differed strongly by cytological category.

### 2.5. Data Validation and Quality Control

All age-by-cytology cells were checked for arithmetic consistency among the cytology total, HPV-tested total, HPV-positive and HPV-negative totals, single and multiple totals, and seven mutually exclusive infection-pattern cells. One deterministic inconsistency affected the primary endpoint: for age 20/ASC-US, the recorded multiple-infection total was 0, while the four mutually exclusive multiple-pattern cells summed to 3. The primary analysis therefore used the author-approved deterministic sum of 3. A sensitivity analysis retained the recorded value of 0.

The initially supplied age-19/LSIL pattern cells were replaced with the author-verified source table: HPV16 + HPV18, 2; HPV16 + other hrHPV, 0; HPV18 + other hrHPV, 0; and positivity in all three reportable categories, 1, yielding 3 multiple infections. The age-25/NILM pattern cells were likewise replaced with the author-verified values of 1 for each of the four multiple-infection combinations, yielding 4 multiple infections. Following these corrections, all marginal genotype counts, mutually exclusive pattern cells, and HPV-positive totals reconciled arithmetically. The original source file was preserved unchanged, and every analytical adjustment was logged.

### 2.6. Statistical Analysis

Counts and percentages were summarized by cytology and single-year age. Wilson score intervals were used for binomial 95% confidence intervals (CIs). Pearson chi-square tests compared HPV testing, HPV positivity among tested women, and multiple infection among HPV-positive women across cytological categories.

Grouped binomial logistic regression was fitted to aggregated success/failure counts. For HPV positivity, predictors were cytology (reference: NILM) and age as an eight-level categorical variable (reference: 18 years). For multiple infections among HPV-positive women, the same predictors were used. Overall effects of cytology and age were evaluated with likelihood-ratio tests comparing nested models. Adjusted odds ratios (aORs) and 95% CIs were derived from maximum-likelihood coefficients. A complementary model treated centered age as a continuous variable for the multiple-infection endpoint.

Age was evaluated primarily as an eight-level categorical variable to avoid imposing a linear relationship over the narrow 18–25-year interval and to display the observed age-specific pattern. As a secondary summary, age was dichotomized at the observed median of 22 years, yielding nearly balanced groups aged 18–22 and 23–25 years. Crude proportions were compared using Pearson chi-square and Fisher’s exact tests. Additive grouped-binomial models produced common cytology-adjusted age-group estimates, and the age-group-by-cytology interaction was tested by likelihood-ratio comparison. Because the interactions were statistically significant, cytology-stratified estimates were emphasized, and the common adjusted estimates were treated only as averages across heterogeneous strata. No geographic subgroup analysis was performed; the Žilina region defined the study setting.

Tests were two-sided with α = 0.05. Analyses were performed in Python 3.12.13 using NumPy 2.3.5, pandas 2.2.3, and SciPy 1.17.0; regression estimates were independently reproduced with scikit-learn 1.8.0. Figures were generated with Matplotlib 3.10.8. Given aggregate input data, no individual-level confounder adjustment or cluster-robust analysis was possible.

## 3. Results

### 3.1. Analytical Cohort and HPV-Testing Context

Of 7574 eligible women with NILM/ASC-US/LSIL, 804 (10.6%) had an HPV DNA result. The testing fraction differed markedly by cytology: 396/7050 (5.6%) for NILM, 243/312 (77.9%) for ASC-US, and 165/212 (77.8%) for LSIL (*p* < 0.001). NILM women underwent testing at their own request during preventive examinations, whereas testing in ASC-US and LSIL occurred in the context of abnormal cytology. Accordingly, the tested NILM subgroup was used to estimate opportunistic prevalence; positivity in abnormal cytology and in the combined tested cohort represents diagnostic or triage yield rather than general-population prevalence.

### 3.2. High-Risk HPV Prevalence in NILM and Positivity by Cytology

Among 396 cytologically negative women who elected HPV testing during preventive examinations, 75 were hrHPV-positive, corresponding to an opportunistic prevalence of 18.9% (95% CI, 15.4–23.1%) in the tested NILM subgroup. Among the 804 tested women across all three cytological categories, 295 were hrHPV-positive (36.7%; 95% CI, 33.4–40.1%). Positivity increased from 18.9% in NILM to 47.7% in ASC-US and 63.0% in LSIL (*p* < 0.001; Table 1). After adjustment for single-year age, HPV positivity remained associated with cytology (overall *p* < 0.001; Table 2). Compared with NILM, the aOR was 4.52 (95% CI, 3.09–6.61; *p* < 0.001) for ASC-US and 8.83 (95% CI, 5.73–13.62; *p* < 0.001) for LSIL.

**Figure 2:**
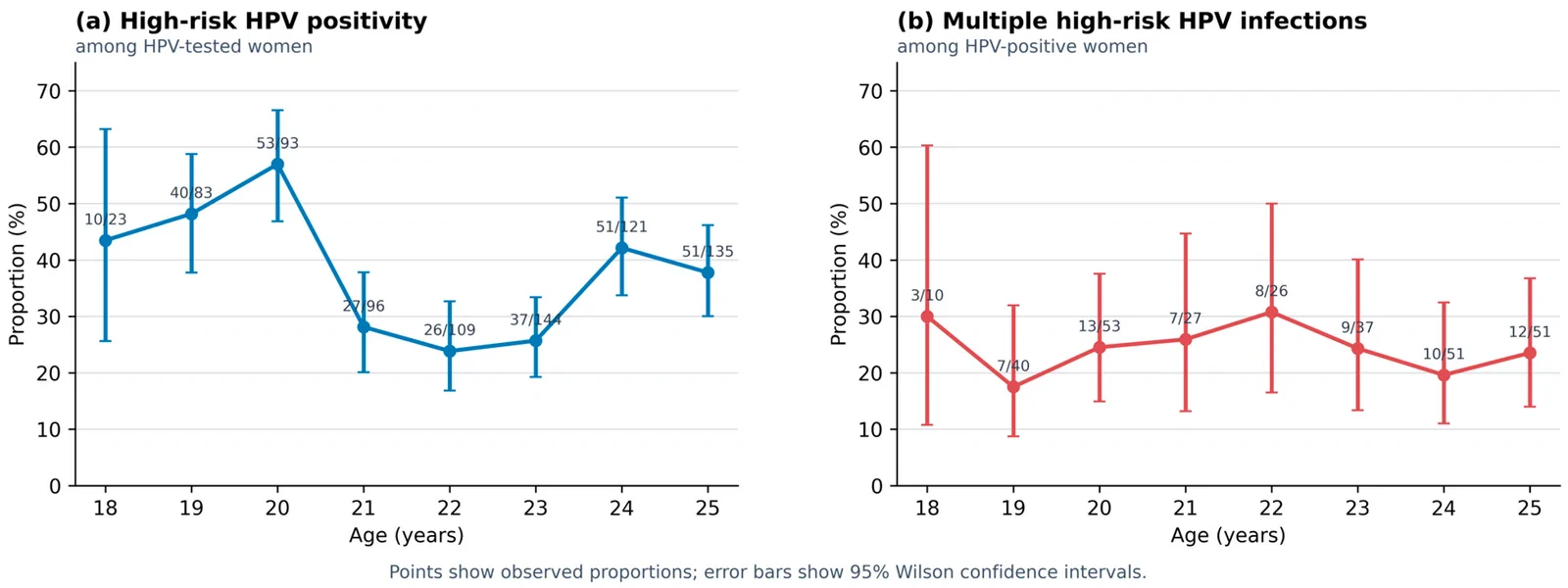
Age-specific outcomes with Wilson 95% confidence intervals. (**a**) High-risk HPV positivity among HPV-tested women. (**b**) Multiple high-risk HPV infections among HPV-positive women. Point estimates are connected only to aid visual interpretation; the study is cross-sectional, and the age pattern should not be interpreted as longitudinal change.

Age-specific positivity showed no consistent trend: it was highest at age 20 (57.0%), fell at ages 21–23 (23.9–28.1%), and increased at ages 24–25 (37.8–42.1%; Table 3 and Figure 2a). Single-year age remained associated with positivity after adjustment for cytology (overall *p* < 0.001; Table 2), but the shape of the pattern and selective testing preclude a simple biological age-gradient interpretation.

### 3.3. Secondary Comparison at the Median Age

Among the 804 HPV-tested women, the median age was 22 years (interquartile range, 21–24 years). The median-based comparison included 404 women aged 18–22 and 400 aged 23–25. HPV positivity was 156/404 (38.6%; 95% CI, 34.0–43.4%) and 139/400 (34.8%; 95% CI, 30.2–39.5%), respectively, with no statistically significant crude difference (OR, 0.85; 95% CI, 0.64–1.13; Pearson *p* = 0.256; Fisher’s exact *p* = 0.273; Table 4). An additive model yielded a common cytology-adjusted OR of 0.78 (95% CI, 0.57–1.06; *p* = 0.117), but the age-group-by-cytology interaction was statistically significant (*p* < 0.001). Thus, the common estimate was not interpreted as a uniform age effect; cytology-specific results are provided in Supplementary File S1.

Multiple hrHPV infections occurred in 38/156 HPV-positive women aged 18–22 (24.4%; 95% CI, 18.3–31.7%) and 31/139 aged 23–25 (22.3%; 95% CI, 16.2–29.9%). The crude comparison was not statistically significant (OR, 0.89; 95% CI, 0.52–1.53; Pearson *p* = 0.677; Fisher’s exact *p* = 0.783). The common cytology-adjusted OR was 0.93 (95% CI, 0.53–1.63; *p* = 0.806), while the interaction test indicated heterogeneity by cytology (*p* = 0.006).

Within the electively tested NILM subgroup, hrHPV positivity was 55/207 (26.6%; 95% CI, 21.0–33.0%) at ages 18–22 and 20/189 (10.6%; 95% CI, 7.0–15.8%) at ages 23–25 (OR, 0.33; 95% CI, 0.19–0.57; Pearson *p* < 0.001; Fisher’s exact *p* < 0.001; Table 5). Multiple infections were observed in 12/55 (21.8%; 95% CI, 12.9–34.4%) and 8/20 (40.0%; 95% CI, 21.9–61.3%) HPV-positive women, respectively; this difference was imprecise and not statistically significant (OR, 2.39; 95% CI, 0.80–7.18; Fisher’s exact *p* = 0.144).

### 3.4. Multiple High-Risk HPV Infections

Sixty-nine of 295 hrHPV-positive women met the operational definition of multiple hrHPV infection (23.4%; 95% CI, 18.9–28.5%); 226 (76.6%) had a single reportable positive category. The proportion with multiple hrHPV infection was 26.7% in NILM, 20.7% in ASC-US, and 24.0% in LSIL (*p* = 0.623). After adjustment for age, there was no evidence of an association between cytology and multiple hrHPV infection (overall *p* = 0.471; Table 2). Relative to NILM, the aOR was 0.63 (95% CI, 0.30–1.32; *p* = 0.222) for ASC-US and 0.78 (95% CI, 0.37–1.63; *p* = 0.503) for LSIL.

The proportion with multiple hrHPV infection by single-year age ranged from 17.5% at age 19 to 30.8% at age 22 (Table 3; Figure 2b). Single-year age was not associated with multiple hrHPV infection after adjustment for cytology (overall *p* = 0.891; Table 2). When age was modeled continuously, the aOR per additional year was 1.01 (95% CI, 0.89–1.15; *p* = 0.843).

### 3.5. Infection Patterns

Among 226 women positive in only one cobas reportable category, HPV18 only accounted for 81/226 (35.8%), pooled other hrHPV only for 74/226 (32.7%), and HPV16 only for 71/226 (31.4%). These three reportable results were similarly distributed. Importantly, an other-hrHPV-only result is not proof of a single-genotype infection because the pooled category may contain one or more of 12 genotypes. Among 69 women positive in multiple reportable categories, HPV16 + other hrHPV was most frequent (30/69; 43.5%), followed by HPV16 + HPV18 (17/69; 24.6%), HPV18 + other hrHPV (14/69; 20.3%), and all three categories (8/69; 11.6%; Table 6).

## 4. Discussion

### 4.1. Principal Findings

The primary endpoint showed that 69/295 (23.4%) hrHPV-positive women were positive in at least two cobas reportable categories. The proportion with multiple hrHPV infection did not vary significantly by single-year age or cytological category in the primary models, and the balanced median split showed similar overall proportions at ages 18–22 and 23–25 (24.4% vs. 22.3%). However, the significant age-group-by-cytology interaction indicated that the age-group contrasts differed by cytology and should not be summarized as a single uniform effect. HPV16 combined with pooled other hrHPV was the most frequent pattern of multiple infection. In the secondary NILM preventive subgroup, 75/396 (18.9%) were hrHPV-positive, with lower positivity at ages 23–25 than 18–22 (10.6% vs. 26.6%).

The absence of a statistically detectable association between multiple hrHPV infection and cytology should be interpreted narrowly and is not evidence of equivalence among NILM, ASC-US, and LSIL. The aggregate dataset did not permit a study-specific blinded cytology re-review or interobserver-reproducibility assessment; cytology-stratified findings are therefore exploratory and hypothesis-generating. They do not establish equal oncogenic potential and do not address histological CIN2+, persistence, clearance, or progression.

### 4.2. Comparison with Previous Evidence

The observed 23.4% proportion of multiple hrHPV infections lies between estimates from studies using broader genotyping. Dickson et al. reported multiple types in 19.0% of HPV-positive samples in a cohort of more than 309,000 women, whereas Chaturvedi et al. reported 43.2% in a research assay detecting 25 types [6,7]. A 2024 systematic review and meta-analysis also documented substantial variation by age, region, lesion category, and laboratory method [13]. These comparisons show that the reported frequency of multiple infections depends strongly on the number of detectable types and the study denominator.

The preceding Northern Slovak study found 37.92% hrHPV positivity in the combined 18–25-year tested cohort and 8.20% among NILM women across all ages in 2023 [1]. Using the same partial-genotyping platform, pooled other hrHPV was the most frequently reported category (17.73% of tested women), followed by HPV16 (6.03%) and HPV18 (1.57%) [1]. These overlapping category-specific proportions do not resolve individual non-16/18 genotypes and cannot define a national Slovak genotype profile. The prior and current positivity estimates are not directly interchangeable because age structure, testing uptake, cytology inclusion, and testing indications differed.

Recent clinical studies likewise show that multiple infection is not consistently associated with lesion severity. Koç et al. reported a multiple-type proportion near 25% and did not find a monotonic increase across histological severity [14]. Baek et al. emphasized the dominant oncogenic potential of HPV16 single-type infections [15], while a large 2026 Greek study found more multiple infections in younger women and higher odds of ASC-US/LSIL, but the opposite direction for HSIL [16]. Together with population-based analyses showing little evidence of genotype synergy [7,8], these data support genotype-specific rather than count-only risk interpretation.

Direct comparisons within the same age range remain assay- and design-dependent. Among 208 unvaccinated sexually active women aged 18–25 years in Paraguay, Bobadilla et al. reported hrHPV positivity of 42.3% and multiple-type infection in 43.0% of HPV-positive samples using an assay resolving 35 genotypes [17]. These estimates are not directly interchangeable with ours because recruitment, vaccination status, cytological composition, denominator, and genotyping resolution differed. In the present cohort, single reportable-category results were nearly evenly distributed among HPV18 only, pooled other hrHPV only, and HPV16 only. The review by Ye et al. demonstrates that genotype distributions vary by lesion grade and histology, with HPV16 prominent in severe squamous lesions and HPV18 relatively enriched in glandular disease [18]. Our assay-specific categories should therefore not be interpreted as an extended-genotype profile of the Slovak population.

### 4.3. Age, Vaccination, and Screening Context

The single-year hrHPV positivity curve across all tested cytological categories showed no consistent age-related trend. In the balanced median split, overall positivity was 38.6% at ages 18–22 and 34.8% at ages 23–25, with no significant crude difference. A significant age-group-by-cytology interaction indicated that the contrast differed among NILM, ASC-US, and LSIL, reinforcing the primacy of the full categorical-age analysis. Within electively tested NILM women, positivity declined from 26.6% to 10.6%; this contrast remains vulnerable to age-related differences in voluntary testing, vaccination, and unmeasured behavior. These data describe a selected preventive cohort and do not support a nationally representative age-specific prevalence curve or screening below the national eligibility age.

Vaccination status is an important missing determinant in a cohort born approximately between 1999 and 2006. In the Costa Rica HPV Vaccine Trial, Sierra et al. followed HPV16/18-positive women aged 18–25 without cytological HSIL and reported high 48-month clearance probabilities but non-zero cumulative CIN2+ and CIN3+ risk, underscoring that cross-sectional detection cannot determine persistence or progression [19]. Contemporary vaccinated Danish cohorts also show profound reductions in HPV16/18 detection [20]. Without person-level vaccination histories, the present study cannot determine whether age-specific patterns reflect vaccine uptake, sexual exposure, or referral behavior.

### 4.4. Strengths and Limitations

Strengths include the focused analysis of an understudied young age range, complete stratification by single-year age and the three most frequent low-grade cytological categories, exact enumeration of mutually exclusive reportable-category patterns, age-adjusted grouped-binomial models, and a documented sensitivity analysis for the principal source inconsistency. Separating the HPV-positivity denominator from the multiple-infection denominator also avoids a common interpretive error.

The study has several important limitations. First, NILM testing was voluntary: only 396/7050 women with NILM were tested, and this regional convenience sample cannot establish nationally representative prevalence. Positivity in ASC-US/LSIL and in the combined tested cohort is test yield. Second, aggregate counts did not permit adjustment for vaccination, smoking, sexual history, parity, immunosuppression, or socioeconomic factors. Both LBC and conventional cytology were used, but the preparation method was unavailable for individual women. Although single-laboratory processing and standardized Bethesda reporting supported routine consistency, no study-specific blinded re-review or interobserver-reproducibility assessment was available. Misclassification among NILM, ASC-US, and LSIL cannot be excluded; cytology-stratified comparisons are therefore exploratory and hypothesis-generating rather than evidence of equivalence.

Third, the cobas assay provides partial rather than extended genotyping; the pooled other-hrHPV result may conceal multiple types, so the operational estimate may underestimate infection with multiple individual genotypes.

### 4.5. Implications and Future Research

For surveillance, the present findings support reporting multiple hrHPV infection as an assay-specific laboratory pattern and always stating its denominator. Clinically, the presence of multiple hrHPV infections should not be treated as a substitute for genotype-specific risk, cytology, persistence, or histology. Future work should use patient-level identifiers, vaccination linkage, extended genotyping, and longitudinal follow-up to determine whether specific combinations alter clearance or CIN2+ risk in young Slovak women. A prespecified comparison with the 2023 cohort could then assess genuine temporal change after harmonizing cytology inclusion, testing indications, and denominators.

## 5. Conclusions

In this regional cohort of women aged 18–25 years with NILM, ASC-US, or LSIL cytology, 23.4% of hrHPV-positive women had concurrent positivity in at least two cobas reportable categories. HPV16 combined with pooled other hrHPV was the most frequent pattern of multiple infection. The primary single-year models did not detect an association between multiple hrHPV infection and age or cytological category. The proportion of women with multiple hrHPV infection was also similar between the two median-based age groups, although the age-group association differed across cytological strata and should be interpreted cautiously. Among electively tested women with NILM, hrHPV positivity was 18.9% and was lower at ages 23–25 than at ages 18–22. Because testing was voluntary and the assay provided only partial genotyping, these findings describe a selected regional cohort and cannot be generalized to population prevalence or used to infer infection with multiple individual genotypes, persistence, or progression.

## Figures and Tables

**Table 1:** Study population, HPV testing, HPV positivity, and multiple infections by cytology.

Cytology	All Women, *n*	HPV Tested, *n* (% of Women)	HPV-Positive, *n* (% Tested; 95% CI)	Multiple hrHPV Infection, *n* (% HPV-Positive; 95% CI)
NILM	7050	396 (5.6)	75 (18.9; 15.4–23.1)	20 (26.7; 18.0–37.6)
ASC-US	312	243 (77.9)	116 (47.7; 41.5–54.0)	24 (20.7; 14.3–28.9)
LSIL	212	165 (77.8)	104 (63.0; 55.4–70.0)	25 (24.0; 16.8–33.1)
Total	7574	804 (10.6)	295 (36.7; 33.4–40.1)	69 (23.4; 18.9–28.5)

**Note.** Percentages use the denominator named in each column. CI, confidence interval; HPV, human papillomavirus. Other abbreviations are defined in Figure 1.

**Table 2:** Adjusted associations of HPV positivity and multiple hrHPV infection with cytology and single-year age.

Outcome	Adjusted Association	aOR	95% CI	*p*-Value
HPV positivity	Overall cytology association	—	—	<0.001
	ASC-US vs. NILM	4.52	3.09–6.61	<0.001
	LSIL vs. NILM	8.83	5.73–13.62	<0.001
	Overall single-year age association	—	—	<0.001
Multiple hrHPV infection	Overall cytology association	—	—	0.471
	ASC-US vs. NILM	0.63	0.30–1.32	0.222
	LSIL vs. NILM	0.78	0.37–1.63	0.503
	Overall single-year age association	—	—	0.891

Note. Overall *p*-values assess cytology or categorical single-year age as a whole in models containing both predictors. Odds ratios for ASC-US and LSIL are relative to NILM and adjusted for categorical single-year age. Dashes indicate that no single odds ratio applies to an overall multi-category association. The complementary continuous-age model is reported in Section 3.4 and Supplementary File S1. aOR, adjusted odds ratio; CI, confidence interval.

**Table 3:** Age-specific HPV positivity and multiple high-risk HPV infections.

Age, Years	Women, *n*	HPV Tested, *n*	HPV-Positive, *n* (%; 95% CI)	Multiple hrHPV Infection, *n* (% HPV-Positive; 95% CI)
18	507	23	10 (43.5; 25.6–63.2)	3 (30.0; 10.8–60.3)
19	683	83	40 (48.2; 37.8–58.8)	7 (17.5; 8.7–31.9)
20	803	93	53 (57.0; 46.8–66.6)	13 (24.5; 14.9–37.6)
21	950	96	27 (28.1; 20.1–37.8)	7 (25.9; 13.2–44.7)
22	901	109	26 (23.9; 16.8–32.7)	8 (30.8; 16.5–50.0)
23	1101	144	37 (25.7; 19.3–33.4)	9 (24.3; 13.4–40.1)
24	1203	121	51 (42.1; 33.7–51.1)	10 (19.6; 11.0–32.5)
25	1426	135	51 (37.8; 30.0–46.2)	12 (23.5; 14.0–36.8)
Total	7574	804	295 (36.7; 33.4–40.1)	69 (23.4; 18.9–28.5)

**Note.** HPV-positive percentages use HPV-tested women as the denominator. Multiple-infection percentages use HPV-positive women as the denominator.

**Table 4:** Secondary comparison using the observed median age of 22 years.

Age, Years	HPV Tested, *n*	HPV-Positive, *n* (%; 95% CI)	Multiple hrHPV, *n* (% HPV-Positive; 95% CI)	Crude HPV-Positive OR (95% CI); *p*	Crude Multiple-Infection OR (95% CI); *p*
18–22	404	156 (38.6; 34.0–43.4)	38 (24.4; 18.3–31.7)	Reference	Reference
23–25	400	139 (34.8; 30.2–39.5)	31 (22.3; 16.2–29.9)	0.85 (0.64–1.13); 0.256	0.89 (0.52–1.53); 0.677

Note. Odds ratios compare ages 23–25 with ages 18–22 and are unadjusted. Pearson *p*-values are shown in the table; Fisher’s exact *p*-values were 0.273 for HPV positivity and 0.783 for multiple infection. Additive common cytology-adjusted estimates and interaction tests are reported in the text and Supplementary File S1. CI, confidence interval; OR, odds ratio.

**Table 5:** Median-based age comparison in the electively tested NILM preventive subgroup.

Age, Years	HPV Tested, *n*	hrHPV-Positive, *n* (%; 95% CI)	Multiple hrHPV, *n* (% HPV-Positive; 95% CI)	hrHPV OR (95% CI); *p*	Multiple-Infection OR (95% CI); *p*
18–22	207	55 (26.6; 21.0–33.0)	12 (21.8; 12.9–34.4)	Reference	Reference
23–25	189	20 (10.6; 7.0–15.8)	8 (40.0; 21.9–61.3)	0.33 (0.19–0.57); <0.001	2.39 (0.80–7.18); 0.144

Note. NILM women elected HPV testing during preventive examinations. Overall hrHPV positivity in this subgroup was 75/396 (18.9%; 95% CI, 15.4–23.1%). ORs compare ages 23–25 with ages 18–22. This regional voluntary-testing sample is not nationally representative. CI, confidence interval; OR, odds ratio.

**Table 6:** Single- and multiple-category high-risk HPV patterns, with percentages calculated within the relevant pattern group.

Pattern	*n*	% Within Pattern Group	Pattern Group (Denominator)
HPV16 only	71	31.4	Single category (*n* = 226)
HPV18 only	81	35.8	Single category (*n* = 226)
Other hrHPV only	74	32.7	Single category (*n* = 226)
HPV16 + HPV18	17	24.6	Multiple categories (*n* = 69)
HPV16 + other hrHPV	30	43.5	Multiple categories (*n* = 69)
HPV18 + other hrHPV	14	20.3	Multiple categories (*n* = 69)
HPV16 + HPV18 + other hrHPV	8	11.6	Multiple categories (*n* = 69)
All HPV-positive	295	100.0	All positive (*n* = 295)

Note. Percentages for single-category results use 226 as the denominator; percentages for multiple-category patterns use 69. ‘Other hrHPV’ is a pooled result containing 12 high-risk types and may conceal infection with multiple individual genotypes.

## Data Availability

The aggregated analytical data and statistical outputs supporting the reported results are provided in Supplementary File S1, and the reproducible analysis code is provided in Supplementary File S2. The underlying routine laboratory records are not publicly available because of institutional data-governance and privacy restrictions.

## Supplementary Materials

The following supporting information can be downloaded at: https://www.mdpi.com/article/10.3390/pathogens15090949/s1, Supplementary File S1: Aggregated analytical workbook containing the analysis cells, cytology and age summaries, infection patterns, model outputs, and quality-control log; Supplementary File S2: Reproducible Python analysis code.

## Abbreviations

Abbrev.	Definition
AGC	Atypical glandular cells
aOR	Adjusted odds ratio
ASC-H	Atypical squamous cells—cannot exclude HSIL
ASC-US	Atypical squamous cells of undetermined significance
CI	Confidence interval
CIN	Cervical intraepithelial neoplasia
HPV	Human papillomavirus
hrHPV	High-risk human papillomavirus
HSIL	High-grade squamous intraepithelial lesion
LSIL	Low-grade squamous intraepithelial lesion
NILM	Negative for intraepithelial lesion or malignancy
STROBE	Strengthening the Reporting of Observational Studies in Epidemiology
