# Supplementary material for: Multiple High-Risk HPV Infections and Opportunistic HPV Prevalence Among Women Aged 18–25 Years with NILM, ASC-US, or LSIL Cytology: A 2024 Cross-Sectional Study
Source: Pathogens. 2026 Sep 7;15(9):949. doi: 10.3390/pathogens15090949 (PMC13610253; doi:10.3390/pathogens15090949)
Supplement: Supplementary file 1 [file pathogens-15-00949-s001.zip › Supplementary_File_S2_HPV_2024_Reproducible_Code.pdf]

Supplementary File S2: Reproducible Python Analysis Code

Multiple High-Risk HPV Infections and Opportunistic HPV Prevalence among Women Aged 18-25 Years with NILM, ASC-US, or LSIL Cytology: A 2024 Cross-Sectional Study

This document provides the complete Python source code used for the statistical analyses and for generating Figures 1-2. The authoritative aggregate analytical tables are provided separately in Supplementary File S1.

1. Scope and analytical workflow

The analysis describes high-risk human papillomavirus (hrHPV) positivity and multiple hrHPV infections among women aged 18-25 years with NILM, ASC-US, or LSIL cytology. The first script validates aggregate counts, applies the documented corrections, calculates descriptive statistics and grouped-binomial models, and creates the study figures. The second script performs the secondary median-based comparison of ages 18-22 versus 23-25 years, including cytology-stratified estimates and interaction tests.

2. Software environment

Component	Version / specification
Python	3.12.13
NumPy	2.3.5
pandas	2.2.3
SciPy	1.17.0
Matplotlib	3.10.8
scikit-learn	1.8.0 (independent reproduction of regression estimates)

3. Reproduction instructions

Save Code Listing A as `work/analyze_hpv.py` and Code Listing B as `work/compare_median_age_groups.py`. Provide the aggregate input table in the path expected by the scripts, install the software versions listed above, and run:

```
python work/analyze_hpv.py
python work/compare_median_age_groups.py
```

Outputs are written to `work/analysis/` and `work/figures/`. The scripts include arithmetic quality-control checks and stop if the prespecified totals do not reconcile.

4. Source-code integrity

File	SHA-256
analyze_hpv.py	b20784ec6ecb21076ca07cf0755ef2868a5e0e1f36b76c8b9da3b0eb897c6582
compare_median_age_groups.py	0bddd22ac42ac3a32080162eafe5fec825d123255297bf42c2acae3db051b901

5. Key verification outputs

The table below records the principal median-based results reproduced by Code Listing B. Full analytical outputs and denominators are supplied in Supplementary File S1.

Outcome	Ages 18-22	Ages 23-25	Effect estimate	p-value
hrHPV positivity, all tested	156/404 (38.6%)	139/400 (34.8%)	OR 0.85 (95% CI 0.64-1.13)	0.256
Multiple hrHPV infection	38/156 (24.4%)	31/139 (22.3%)	OR 0.89 (95% CI 0.52-1.53)	0.677
hrHPV positivity, NILM subgroup	55/207 (26.6%)	20/189 (10.6%)	OR 0.33 (95% CI 0.19-0.57)	<0.001
Multiple infection, NILM subgroup	12/55 (21.8%)	8/20 (40.0%)	OR 2.39 (95% CI 0.80-7.18)	0.144

ORs compare ages 23-25 with ages 18-22. The first two p-values are Pearson chi-square p-values; the NILM subgroup p-values are Fisher exact p-values. CI, confidence interval; OR, odds ratio.

Interpretation note: The median-based comparison is a secondary analysis. Because the age-group-by-cytology interaction was statistically significant for both overall hrHPV positivity and multiple hrHPV infection, common cytology-adjusted estimates should be interpreted only as averages across heterogeneous cytological strata.

Code Listing A. analyze_hpv.py

Primary validation, descriptive analyses, grouped-binomial models, and figure generation.

```
import json
import math
from pathlib import Path

import numpy as np
import pandas as pd
from scipy.optimize import minimize
from scipy.special import expit
from scipy.stats import chi2, chi2_contingency, norm

import matplotlib
matplotlib.use("Agg")
import matplotlib.pyplot as plt
from matplotlib.patches import FancyBboxPatch


ROOT = Path(__file__).resolve().parents[1]
SOURCE_JSON = ROOT / "work/source_01_H_rok1.json"
OUT = ROOT / "work/analysis"
FIG = ROOT / "work/figures"
OUT.mkdir(parents=True, exist_ok=True)
FIG.mkdir(parents=True, exist_ok=True)


def wilson_ci(x, n, alpha=0.05):
    if n == 0:
        return (math.nan, math.nan)
    z = norm.ppf(1 - alpha / 2)
    p = x / n
    den = 1 + z * z / n
    ctr = (p + z * z / (2 * n)) / den
    half = z * math.sqrt(p * (1 - p) / n + z * z / (4 * n * n)) / den
    return ctr - half, ctr + half


def cochran_armitage(success, total, scores):
    success = np.asarray(success, dtype=float)
    total = np.asarray(total, dtype=float)
    scores = np.asarray(scores, dtype=float)
    p_bar = success.sum() / total.sum()
    score_bar = np.sum(total * scores) / total.sum()
    numerator = np.sum(scores * (success - total * p_bar))
    denominator = math.sqrt(p_bar * (1 - p_bar) * np.sum(total * (scores - score_bar) ** 2))
    z = numerator / denominator
    p = 2 * norm.sf(abs(z))
    return float(z), float(p)


def fit_grouped_logistic(df, outcome, total, terms, reference_levels=None):
    reference_levels = reference_levels or {}
    cols = [np.ones(len(df))]
    names = ["Intercept"]
    for term in terms:
        if term.startswith("C(") and term.endswith(")"):
            var = term[2:-1]
            levels = sorted(df[var].unique().tolist())
            ref = reference_levels.get(var, levels[0])
            for level in levels:
                if level == ref:
                    continue
                cols.append((df[var].to_numpy() == level).astype(float))
                names.append(f"{var}[{level}]")
```

```
        else:
            cols.append(df[term].to_numpy(dtype=float))
            names.append(term)
    X = np.column_stack(cols)
    y = df[outcome].to_numpy(dtype=float)
    n = df[total].to_numpy(dtype=float)

    def nll(beta):
        eta = X @ beta
        return float(np.sum(n * np.logaddexp(0, eta) - y * eta))

    def grad(beta):
        eta = X @ beta
        p = expit(eta)
        return X.T @ (n * p - y)

    res = minimize(nll, np.zeros(X.shape[1]), jac=grad, method="BFGS", options={"gtol": 1e-10, "maxiter": 5000})
    beta = res.x
    p = expit(X @ beta)
    info = X.T @ ((n * p * (1 - p))[:, None] * X)
    cov = np.linalg.pinv(info)
    se = np.sqrt(np.diag(cov))
    z = beta / se
    pvals = 2 * norm.sf(np.abs(z))
    coef = pd.DataFrame({
        "term": names,
        "estimate_log_odds": beta,
        "standard_error": se,
        "odds_ratio": np.exp(beta),
        "ci95_low": np.exp(beta - 1.96 * se),
        "ci95_high": np.exp(beta + 1.96 * se),
        "p_value": pvals,
    })
    return {"result": res, "loglike": -nll(beta), "df_model": X.shape[1] - 1, "coefficients": coef}


def lr_test(full, reduced):
    statistic = 2 * (full["loglike"] - reduced["loglike"])
    degrees = full["df_model"] - reduced["df_model"]
    return {"lr_chi2": statistic, "df": degrees, "p_value": chi2.sf(statistic, degrees)}


with SOURCE_JSON.open("r", encoding="utf-8") as f:
    source = json.load(f)
values = source["values"]

ages = list(range(18, 26))
cytologies = ["NILM", "ASC-US", "LSIL"]
source_label = {"NILM": "NILM", "ASC-US": "ASCUS", "LSIL": "LSIL"}
records = []
qc = []

for age_index, age in enumerate(ages):
    block_start = age_index * 10
    for row_offset in (2, 3, 4):
        row = values[block_start + row_offset]
        cytology = {"NILM": "NILM", "ASCUS": "ASC-US", "LSIL": "LSIL"}[row[0]]
        tests, positive, negative = map(int, row[1:4])
        hpv16, hpv18, other = map(int, row[4:7])
        combinations = [int(float(v)) for v in row[7:11]]
        multi_recorded = int(row[11])
        # Author-supplied correction from the verified 19-year 2024 source table.
        if age == 19 and cytology == "LSIL":
            original_combinations = combinations.copy()
            original_multi = multi_recorded
            combinations = [2, 0, 0, 1]
            multi_recorded = 3
```

```
            qc.append({
                "age": age,
                "cytology": cytology,
                "field": "infection_pattern_cells",
                "recorded": f"{original_combinations}; multiple={original_multi}",
                "derived": f"{combinations}; multiple={multi_recorded}",
                "action": "Replaced with the author-verified 2024 age-19 source table",
            })
        # Author-supplied correction from the verified 25-year 2024 source table.
        if age == 25 and cytology == "NILM":
            original_combinations = combinations.copy()
            combinations = [1, 1, 1, 1]
            qc.append({
                "age": age,
                "cytology": cytology,
                "field": "infection_pattern_cells",
                "recorded": f"{original_combinations}; multiple={multi_recorded}",
                "derived": f"{combinations}; multiple={multi_recorded}",
                "action": "Replaced with the author-verified 2024 age-25 source table",
            })
        multi_derived = sum(combinations)
        multi_main = multi_derived
        if multi_recorded != multi_derived:
            qc.append({
                "age": age,
                "cytology": cytology,
                "field": "multiple_infections",
                "recorded": multi_recorded,
                "derived": multi_derived,
                "action": "Used deterministic sum of the four mutually exclusive combination cells in the primary analysis",
            })
        single = positive - multi_main
        records.append({
            "age": age,
            "cytology": cytology,
            "tested": tests,
            "hpv_positive": positive,
            "hpv_negative": negative,
            "hpv16_present": hpv16,
            "hpv18_present": hpv18,
            "other_hrhpv_present": other,
            "hpv16_hpv18": combinations[0],
            "hpv16_other": combinations[1],
            "hpv18_other": combinations[2],
            "triple": combinations[3],
            "multiple_recorded": multi_recorded,
            "multiple_primary": multi_main,
            "single_primary": single,
        })

data = pd.DataFrame(records)

# Cytology source-population counts are in rows 84-91 (zero-based 83-90).
cyto_population = []
for idx, age in enumerate(ages):
    row = values[83 + idx]
    cyto_population.extend([
        {"age": age, "cytology": "NILM", "cytology_count": int(row[1])},
        {"age": age, "cytology": "ASC-US", "cytology_count": int(row[3])},
        {"age": age, "cytology": "LSIL", "cytology_count": int(row[5])},
    ])
population = pd.DataFrame(cyto_population)
data = data.merge(population, on=["age", "cytology"], how="left", validate="one_to_one")

# Internal checks.
data["check_tested"] = data["hpv_positive"] + data["hpv_negative"] - data["tested"]
```

```python
data["check_positive"] = data["single_primary"] + data["multiple_primary"] - data["hpv_positive"]
if not (data["check_tested"].eq(0).all() and data["check_positive"].eq(0).all()):
    raise ValueError("Core denominator checks failed")

# Flag two age-specific marginal genotype inconsistencies without changing source values.
data["single16_implied"] = data["hpv16_present"] - data["hpv16_hpv18"] - data["hpv16_other"] - data["triple"]
data["single18_implied"] = data["hpv18_present"] - data["hpv16_hpv18"] - data["hpv18_other"] - data["triple"]
data["single_other_implied"] = data["other_hrhpv_present"] - data["hpv16_other"] - data["hpv18_other"] - data["triple"]
for _, row in data.iterrows():
    for field in ["single16_implied", "single18_implied", "single_other_implied"]:
        if row[field] < 0:
            qc.append({
                "age": int(row["age"]),
                "cytology": row["cytology"],
                "field": field,
                "recorded": "incompatible marginal count",
                "derived": int(row[field]),
                "action": "Age-specific single-category decomposition not analyzed; pooled categories reconcile exactly",
            })

summary_cytology = data.groupby("cytology", sort=False).agg(
    cytology_count=("cytology_count", "sum"),
    tested=("tested", "sum"),
    hpv_positive=("hpv_positive", "sum"),
    hpv_negative=("hpv_negative", "sum"),
    single=("single_primary", "sum"),
    multiple=("multiple_primary", "sum"),
).reset_index()
summary_cytology["testing_fraction"] = summary_cytology["tested"] / summary_cytology["cytology_count"]
summary_cytology["positivity_fraction"] = summary_cytology["hpv_positive"] / summary_cytology["tested"]
summary_cytology["multiple_among_positive_fraction"] = summary_cytology["multiple"] / summary_cytology["hpv_positive"]

summary_age = data.groupby("age").agg(
    cytology_count=("cytology_count", "sum"),
    tested=("tested", "sum"),
    hpv_positive=("hpv_positive", "sum"),
    hpv_negative=("hpv_negative", "sum"),
    single=("single_primary", "sum"),
    multiple=("multiple_primary", "sum"),
).reset_index()
summary_age["testing_fraction"] = summary_age["tested"] / summary_age["cytology_count"]
summary_age["positivity_fraction"] = summary_age["hpv_positive"] / summary_age["tested"]
summary_age["multiple_among_positive_fraction"] = summary_age["multiple"] / summary_age["hpv_positive"]
summary_age[["positivity_ci_low", "positivity_ci_high"]] = summary_age.apply(
    lambda r: pd.Series(wilson_ci(r["hpv_positive"], r["tested"])), axis=1
)
summary_age[["multiple_ci_low", "multiple_ci_high"]] = summary_age.apply(
    lambda r: pd.Series(wilson_ci(r["multiple"], r["hpv_positive"])), axis=1
)

# Pooled exact infection patterns by cytology; age-specific decomposition was intentionally avoided.
patterns = []
for cytology, g in data.groupby("cytology", sort=False):
    s16 = int(g["hpv16_present"].sum() - g["hpv16_hpv18"].sum() - g["hpv16_other"].sum() - g["triple"].sum())
    s18 = int(g["hpv18_present"].sum() - g["hpv16_hpv18"].sum() - g["hpv18_other"].sum() - g["triple"].sum())
    so = int(g["other_hrhpv_present"].sum() - g["hpv16_other"].sum() - g["hpv18_other"].sum() - g["triple"].sum())
    vals = {
        "HPV16 only": s16,
        "HPV18 only": s18,
        "Other hrHPV only": so,
        "HPV16 + HPV18": int(g["hpv16_hpv18"].sum()),
        "HPV16 + other hrHPV": int(g["hpv16_other"].sum()),
        "HPV18 + other hrHPV": int(g["hpv18_other"].sum()),
        "HPV16 + HPV18 + other hrHPV": int(g["triple"].sum()),
```

```
    }
    if sum(vals.values()) != int(g["hpv_positive"].sum()) or min(vals.values()) < 0:
        raise ValueError(f"Pooled pattern reconciliation failed for {cytology}")
    for pattern, count in vals.items():
        patterns.append({"cytology": cytology, "pattern": pattern, "count": count})
patterns = pd.DataFrame(patterns)
overall_patterns = patterns.groupby("pattern", sort=False)["count"].sum().reset_index()
overall_patterns["fraction_of_positive"] = overall_patterns["count"] / data["hpv_positive"].sum()
overall_patterns["fraction_of_multiple"] = np.where(
    overall_patterns["pattern"].str.contains("+", regex=False),
    overall_patterns["count"] / data["multiple_primary"].sum(),
    np.nan,
)

# Unadjusted contingency tests.
testing_table = np.column_stack([
    summary_cytology["tested"].to_numpy(),
    (summary_cytology["cytology_count"] - summary_cytology["tested"]).to_numpy(),
])
positive_table = np.column_stack([
    summary_cytology["hpv_positive"].to_numpy(),
    summary_cytology["hpv_negative"].to_numpy(),
])
multiple_table = np.column_stack([
    summary_cytology["multiple"].to_numpy(),
    summary_cytology["single"].to_numpy(),
])
tests = {
    "testing_by_cytology": dict(zip(["chi2", "p_value", "df", "expected"], chi2_contingency(testing_table, correction=False))),
    "positivity_by_cytology": dict(zip(["chi2", "p_value", "df", "expected"], chi2_contingency(positive_table, correction=False))),
    "multiple_by_cytology": dict(zip(["chi2", "p_value", "df", "expected"], chi2_contingency(multiple_table, correction=False))),
}
for value in tests.values():
    value["expected"] = np.asarray(value["expected"]).tolist()

# Age-adjusted grouped binomial regression.
refs = {"cytology": "NILM", "age": 18}
positive_full = fit_grouped_logistic(data, "hpv_positive", "tested", ["C(cytology)", "C(age)"], refs)
positive_no_cyt = fit_grouped_logistic(data, "hpv_positive", "tested", ["C(age)"], refs)
positive_no_age = fit_grouped_logistic(data, "hpv_positive", "tested", ["C(cytology)"], refs)
multiple_full = fit_grouped_logistic(data, "multiple_primary", "hpv_positive", ["C(cytology)", "C(age)"], refs)
multiple_no_cyt = fit_grouped_logistic(data, "multiple_primary", "hpv_positive", ["C(age)"], refs)
multiple_no_age = fit_grouped_logistic(data, "multiple_primary", "hpv_positive", ["C(cytology)"], refs)
multiple_cont = fit_grouped_logistic(data.assign(age_centered=data["age"] - 18), "multiple_primary", "hpv_positive", ["C(cytology)", "age_centered"], refs)

model_tests = {
    "positivity_cytology_adjusted_for_age": lr_test(positive_full, positive_no_cyt),
    "positivity_age_adjusted_for_cytology": lr_test(positive_full, positive_no_age),
    "multiple_cytology_adjusted_for_age": lr_test(multiple_full, multiple_no_cyt),
    "multiple_age_adjusted_for_cytology": lr_test(multiple_full, multiple_no_age),
}

age_trend_positive = cochran_armitage(summary_age["hpv_positive"], summary_age["tested"], summary_age["age"])
age_trend_multiple = cochran_armitage(summary_age["multiple"], summary_age["hpv_positive"], summary_age["age"])

# Sensitivity analysis retaining the originally recorded zero for age 20 / ASC-US.
sens = data.copy()
sens["multiple_sensitivity"] = sens["multiple_primary"]
sens.loc[(sens["age"] == 20) & (sens["cytology"] == "ASC-US"), "multiple_sensitivity"] = 0
sens["single_sensitivity"] = sens["hpv_positive"] - sens["multiple_sensitivity"]
sens_summary = sens.groupby("cytology", sort=False).agg(multiple=("multiple_sensitivity", "sum"), single=("single_sensitivity", "sum")).reset_index()
sens_table = np.column_stack([sens_summary["multiple"], sens_summary["single"]])
```

```
sens_chi = chi2_contingency(sens_table, correction=False)
sens_full = fit_grouped_logistic(sens, "multiple_sensitivity", "hpv_positive", ["C(cytology)", "C(age)"], refs)
sens_no_cyt = fit_grouped_logistic(sens, "multiple_sensitivity", "hpv_positive", ["C(age)"], refs)
sens_no_age = fit_grouped_logistic(sens, "multiple_sensitivity", "hpv_positive", ["C(cytology)"], refs)
sensitivity = {
    "total_multiple": int(sens["multiple_sensitivity"].sum()),
    "unadjusted_chi2": float(sens_chi[0]),
    "unadjusted_p": float(sens_chi[1]),
    "adjusted_cytology": lr_test(sens_full, sens_no_cyt),
    "adjusted_age": lr_test(sens_full, sens_no_age),
}

# Key odds ratios for manuscript tables.
coef_positive = positive_full["coefficients"]
coef_multiple = multiple_full["coefficients"]
coef_multiple_cont = multiple_cont["coefficients"]

# Save auditable flat outputs.
data.to_csv(OUT / "analysis_cells.csv", index=False)
population.to_csv(OUT / "source_population_cells.csv", index=False)
summary_cytology.to_csv(OUT / "summary_by_cytology.csv", index=False)
summary_age.to_csv(OUT / "summary_by_age.csv", index=False)
patterns.to_csv(OUT / "infection_patterns_by_cytology.csv", index=False)
overall_patterns.to_csv(OUT / "infection_patterns_overall.csv", index=False)
pd.DataFrame(qc).to_csv(OUT / "quality_control_log.csv", index=False)
coef_positive.to_csv(OUT / "model_hpv_positivity_coefficients.csv", index=False)
coef_multiple.to_csv(OUT / "model_multiple_coefficients.csv", index=False)
coef_multiple_cont.to_csv(OUT / "model_multiple_continuous_age_coefficients.csv", index=False)

results = {
    "totals": {
        "all_cytology_women": 7613,
        "selected_cytology_women": int(population["cytology_count"].sum()),
        "hpv_tested": int(data["tested"].sum()),
        "hpv_positive": int(data["hpv_positive"].sum()),
        "hpv_negative": int(data["hpv_negative"].sum()),
        "single": int(data["single_primary"].sum()),
        "multiple": int(data["multiple_primary"].sum()),
    },
    "contingency_tests": tests,
    "model_likelihood_ratio_tests": model_tests,
    "age_trends": {
        "hpv_positivity": {"z": age_trend_positive[0], "p_value": age_trend_positive[1]},
        "multiple_among_positive": {"z": age_trend_multiple[0], "p_value": age_trend_multiple[1]},
    },
    "sensitivity_recorded_zero": sensitivity,
    "multiple_continuous_age_coefficients": coef_multiple_cont.to_dict(orient="records"),
}
with (OUT / "statistical_results.json").open("w", encoding="utf-8") as f:
    json.dump(results, f, indent=2, ensure_ascii=False, default=float)

bundle = {
    "analysis_cells": data.to_dict(orient="records"),
    "source_population_cells": population.to_dict(orient="records"),
    "summary_by_cytology": summary_cytology.to_dict(orient="records"),
    "summary_by_age": summary_age.to_dict(orient="records"),
    "infection_patterns_by_cytology": patterns.to_dict(orient="records"),
    "infection_patterns_overall": overall_patterns.replace({np.nan: None}).to_dict(orient="records"),
    "quality_control_log": pd.DataFrame(qc).to_dict(orient="records"),
    "model_hpv_positivity_coefficients": coef_positive.to_dict(orient="records"),
    "model_multiple_coefficients": coef_multiple.to_dict(orient="records"),
    "model_multiple_continuous_age_coefficients": coef_multiple_cont.to_dict(orient="records"),
    "statistical_results": results,
}
with (OUT / "analysis_bundle.json").open("w", encoding="utf-8") as f:
    json.dump(bundle, f, indent=2, ensure_ascii=False, default=float)
```

```python
# Figure 1: study flow.
plt.rcParams.update({"font.family": "DejaVu Sans", "font.size": 10})
total_single = int(data["single_primary"].sum())
total_multiple = int(data["multiple_primary"].sum())
total_positive = int(data["hpv_positive"].sum())
fig, ax = plt.subplots(figsize=(8.0, 7.4))
ax.set_xlim(0, 10)
ax.set_ylim(0, 12)
ax.axis("off")
box_fc = "#EFF6FF"
box_ec = "#1F4E79"
green_fc = "#ECFDF5"
gray_fc = "#F3F4F6"

def draw_box(x, y, w, h, title, subtitle, fc=box_fc, ec=box_ec):
    patch = FancyBboxPatch((x, y), w, h, boxstyle="round,pad=0.05,rounding_size=0.08", linewidth=1.5, edgecolor=ec, facecolor=fc)
    ax.add_patch(patch)
    ax.text(x + w / 2, y + h * 0.62, title, ha="center", va="center", fontsize=12, fontweight="bold", color="#0F172A")
    ax.text(x + w / 2, y + h * 0.29, subtitle, ha="center", va="center", fontsize=9.5, color="#334155", wrap=True)

draw_box(2.0, 10.0, 6.0, 1.35, "7,613 unique women", "Women aged 18–25 years examined in 2024")
ax.annotate("", xy=(5, 9.4), xytext=(5, 10.0), arrowprops=dict(arrowstyle="-|>", lw=1.4, color="#64748B"))
draw_box(2.0, 8.05, 6.0, 1.35, "7,574 women included", "NILM: 7,050 | ASC-US: 312 | LSIL: 212", fc=green_fc)
ax.text(8.35, 8.72, "Excluded: 39\nASC-H, HSIL, or AGC", ha="left", va="center", fontsize=9, color="#475569")
ax.annotate("", xy=(5, 7.45), xytext=(5, 8.05), arrowprops=dict(arrowstyle="-|>", lw=1.4, color="#64748B"))
draw_box(2.0, 6.1, 6.0, 1.35, "804 women had an HPV DNA result", "NILM: 396 | ASC-US: 243 | LSIL: 165")
draw_box(0.65, 3.9, 4.0, 1.55, "509 HPV-negative", "63.3% of tested women", fc=gray_fc, ec="#64748B")
draw_box(5.35, 3.9, 4.0, 1.55, "295 HPV-positive", "36.7% of tested women", fc=green_fc)
ax.plot([5, 5], [6.1, 5.75], color="#64748B", lw=1.4)
ax.plot([2.65, 7.35], [5.75, 5.75], color="#64748B", lw=1.4)
ax.annotate("", xy=(2.65, 5.45), xytext=(2.65, 5.75), arrowprops=dict(arrowstyle="-|>", lw=1.4, color="#64748B"))
ax.annotate("", xy=(7.35, 5.45), xytext=(7.35, 5.75), arrowprops=dict(arrowstyle="-|>", lw=1.4, color="#64748B"))
draw_box(
    4.9,
    1.5,
    2.1,
    1.75,
    f"{total_single} single",
    f"{100 * total_single / total_positive:.1f}% of HPV-positive",
    fc="#FFF7ED",
    ec="#C2410C",
)
draw_box(
    7.25,
    1.5,
    2.1,
    1.75,
    f"{total_multiple} multiple",
    f"{100 * total_multiple / total_positive:.1f}% of HPV-positive",
    fc="#FEF2F2",
    ec="#B91C1C",
)
ax.plot([7.35, 7.35], [3.9, 3.6], color="#64748B", lw=1.3)
ax.plot([5.95, 8.3], [3.6, 3.6], color="#64748B", lw=1.3)
ax.annotate("", xy=(5.95, 3.25), xytext=(5.95, 3.6), arrowprops=dict(arrowstyle="-|>", lw=1.3, color="#64748B"))
ax.annotate("", xy=(8.3, 3.25), xytext=(8.3, 3.6), arrowprops=dict(arrowstyle="-|>", lw=1.3, color="#64748B"))
ax.text(5, 0.45, "Analytical outcomes: HPV positivity among tested women and multiple infection among HPV-positive women", ha="center", va="center", fontsize=9, color="#475569")
fig.tight_layout()
fig.savefig(FIG / "Figure_1_study_flow.png", dpi=600, bbox_inches="tight", facecolor="white")
plt.close(fig)

# Figure 2: age-specific outcomes with Wilson intervals.
fig, axes = plt.subplots(1, 2, figsize=(11.5, 4.6), sharex=True)
colors = ["#1F77B4", "#C44E52"]
```

```
for ax, fraction, low, high, title, denominator, color in [
    (axes[0], "positivity_fraction", "positivity_ci_low", "positivity_ci_high", "(a) High-risk HPV positivity", "among HPV-tested women", colors[0]),
    (axes[1], "multiple_among_positive_fraction", "multiple_ci_low", "multiple_ci_high", "(b) Multiple high-risk HPV infections", "among HPV-positive women", colors[1]),
]:
    y = summary_age[fraction].to_numpy() * 100
    yerr = np.vstack([(summary_age[fraction] - summary_age[low]).to_numpy() * 100, (summary_age[high] - summary_age[fraction]).to_numpy() * 100])
    ax.errorbar(summary_age["age"], y, yerr=yerr, fmt="o-", color=color, lw=2, markersize=5.5, capsize=3, capthick=1)
    ax.set_title(title, loc="left", y=1.08, pad=0, fontsize=11.5, fontweight="bold")
    ax.text(0.0, 1.015, denominator, transform=ax.transAxes, ha="left", va="bottom", fontsize=9, color="#475569")
    ax.set_xlabel("Age (years)")
    ax.set_ylabel("Proportion (%)")
    ax.set_xticks(ages)
    ax.set_ylim(0, max(75, math.ceil((y + yerr[1]).max() / 10) * 10))
    ax.grid(axis="y", color="#D1D5DB", lw=0.7, alpha=0.8)
    ax.spines[["top", "right"]].set_visible(False)
    for x, val, num, den in zip(summary_age["age"], y, summary_age["hpv_positive"] if fraction == "positivity_fraction" else summary_age["multiple"], summary_age["tested"] if fraction == "positivity_fraction" else summary_age["hpv_positive"]):
        ax.annotate(f"{int(num)}/{int(den)}", (x, val), xytext=(0, 8), textcoords="offset points", ha="center", fontsize=7.5, color="#374151")
fig.text(0.5, 0.04, "Points show observed proportions; error bars show 95% Wilson confidence intervals.", ha="center", fontsize=9, color="#475569")
fig.subplots_adjust(left=0.08, right=0.98, top=0.82, bottom=0.18, wspace=0.24)
fig.savefig(FIG / "Figure_2_age_specific_outcomes.png", dpi=600, bbox_inches="tight", facecolor="white")
plt.close(fig)

print(json.dumps(results, indent=2, ensure_ascii=False, default=float))
```

Code Listing B. compare_median_age_groups.py

Secondary median-based comparison, cytology-stratified estimates, and interaction testing.

```
import json
from pathlib import Path

import numpy as np
import pandas as pd
from scipy.optimize import minimize
from scipy.special import expit
from scipy.stats import chi2, chi2_contingency, fisher_exact, norm


ROOT = Path(__file__).resolve().parents[1]
CELLS = ROOT / "work/analysis/analysis_cells.csv"
OUT_JSON = ROOT / "work/analysis/age_median_split.json"
OUT_CSV = ROOT / "work/analysis/age_median_split.csv"


def wilson(x, n):
    z = norm.ppf(0.975)
    p = x / n
    den = 1 + z * z / n
    center = (p + z * z / (2 * n)) / den
    half = z * np.sqrt(p * (1 - p) / n + z * z / (4 * n * n)) / den
    return float(center - half), float(center + half)


def weighted_median_age(df):
    by_age = df.groupby("age", as_index=False)["tested"].sum().sort_values("age")
    midpoint = by_age["tested"].sum() / 2
    return int(by_age.loc[by_age["tested"].cumsum() >= midpoint, "age"].iloc[0])


def fit_grouped(
    df, success, total, older_label, include_group=True, include_interaction=False
):
    cols = [np.ones(len(df))]
    names = ["Intercept"]
    for level in ["ASC-US", "LSIL"]:
        cols.append((df["cytology"].to_numpy() == level).astype(float))
        names.append(f"cytology[{level}]")
    if include_group:
        older = (df["age_group"].to_numpy() == older_label).astype(float)
        cols.append(older)
        names.append(f"age_group[{older_label}]")
        if include_interaction:
            for level in ["ASC-US", "LSIL"]:
                cols.append(
                    older * (df["cytology"].to_numpy() == level).astype(float)
                )
                names.append(f"age_group[{older_label}]:cytology[{level}]")
    X = np.column_stack(cols)
    y = df[success].to_numpy(float)
    n = df[total].to_numpy(float)

    def nll(beta):
        eta = X @ beta
        return float(np.sum(n * np.logaddexp(0, eta) - y * eta))

    def grad(beta):
        eta = X @ beta
        return X.T @ (n * expit(eta) - y)

    res = minimize(nll, np.zeros(X.shape[1]), jac=grad, method="BFGS")
```

```
    beta = res.x
    p = expit(X @ beta)
    w = n * p * (1 - p)
    cov = np.linalg.pinv(X.T @ (X * w[:, None]))
    se = np.sqrt(np.diag(cov))
    rows = []
    for name, b, s in zip(names, beta, se):
        rows.append({
            "term": name,
            "estimate": float(b),
            "se": float(s),
            "or": float(np.exp(b)),
            "ci_low": float(np.exp(b - 1.96 * s)),
            "ci_high": float(np.exp(b + 1.96 * s)),
            "p": float(2 * norm.sf(abs(b / s))),
        })
    return {"nll": float(res.fun), "rows": rows}


def lrt(full, reduced, df=1):
    stat = 2 * (reduced["nll"] - full["nll"])
    return {"chi2": float(stat), "df": df, "p": float(chi2.sf(stat, df))}


def odds_ratio_older_vs_younger(table):
    a, b = table[1]
    c, d = table[0]
    estimate = (a * d) / (b * c)
    se = np.sqrt(1 / a + 1 / b + 1 / c + 1 / d)
    return {
        "or": float(estimate),
        "ci_low": float(np.exp(np.log(estimate) - 1.96 * se)),
        "ci_high": float(np.exp(np.log(estimate) + 1.96 * se)),
    }


df = pd.read_csv(CELLS)
median_age = weighted_median_age(df)
younger_label = f"18-{median_age}"
older_label = f"{median_age + 1}-25"
df["age_group"] = np.where(df.age <= median_age, younger_label, older_label)

summary_rows = []
for group, g in df.groupby("age_group", sort=False):
    tested = int(g.tested.sum())
    positive = int(g.hpv_positive.sum())
    multiple = int(g.multiple_primary.sum())
    pos_ci = wilson(positive, tested)
    mult_ci = wilson(multiple, positive)
    summary_rows.append({
        "age_group": group,
        "selected_records": int(g.cytology_count.sum()),
        "tested": tested,
        "hpv_positive": positive,
        "hpv_negative": tested - positive,
        "positivity": positive / tested,
        "positivity_ci_low": pos_ci[0],
        "positivity_ci_high": pos_ci[1],
        "multiple": multiple,
        "single": positive - multiple,
        "multiple_fraction": multiple / positive,
        "multiple_ci_low": mult_ci[0],
        "multiple_ci_high": mult_ci[1],
    })

summary = pd.DataFrame(summary_rows)
young = summary[summary.age_group == younger_label].iloc[0]
```

```
old = summary[summary.age_group == older_label].iloc[0]

pos_table = np.array(
    [[young.hpv_positive, young.hpv_negative], [old.hpv_positive, old.hpv_negative]],
    dtype=int,
)
mult_table = np.array(
    [[young.multiple, young.single], [old.multiple, old.single]], dtype=int
)

pos_chi = chi2_contingency(pos_table, correction=False)
mult_chi = chi2_contingency(mult_table, correction=False)
pos_fisher = fisher_exact(pos_table)
mult_fisher = fisher_exact(mult_table)

pos_full = fit_grouped(df, "hpv_positive", "tested", older_label, True)
pos_reduced = fit_grouped(df, "hpv_positive", "tested", older_label, False)
mult_full = fit_grouped(df, "multiple_primary", "hpv_positive", older_label, True)
mult_reduced = fit_grouped(df, "multiple_primary", "hpv_positive", older_label, False)
pos_interaction = fit_grouped(
    df, "hpv_positive", "tested", older_label, True, include_interaction=True
)
mult_interaction = fit_grouped(
    df,
    "multiple_primary",
    "hpv_positive",
    older_label,
    True,
    include_interaction=True,
)


def group_row(model):
    return next(r for r in model["rows"] if r["term"] == f"age_group[{older_label}]")


result = {
    "comparison": f"{younger_label} years versus {older_label} years",
    "median_age_among_tested": median_age,
    "reference_group": younger_label,
    "summary": summary.to_dict(orient="records"),
    "hpv_positivity": {
        "pearson_chi2": float(pos_chi.statistic),
        "pearson_p": float(pos_chi.pvalue),
        "fisher_or_younger_vs_older": float(pos_fisher.statistic),
        "fisher_p": float(pos_fisher.pvalue),
        "adjusted_older_vs_younger": group_row(pos_full),
        "adjusted_lrt": lrt(pos_full, pos_reduced),
        "age_group_by_cytology_interaction_lrt": lrt(
            pos_interaction, pos_full, df=2
        ),
    },
    "multiple_infection": {
        "pearson_chi2": float(mult_chi.statistic),
        "pearson_p": float(mult_chi.pvalue),
        "fisher_or_younger_vs_older": float(mult_fisher.statistic),
        "fisher_p": float(mult_fisher.pvalue),
        "adjusted_older_vs_younger": group_row(mult_full),
        "adjusted_lrt": lrt(mult_full, mult_reduced),
        "age_group_by_cytology_interaction_lrt": lrt(
            mult_interaction, mult_full, df=2
        ),
    },
}

stratified = []
for cytology, g in df.groupby("cytology", sort=False):
```

```
    y = g[g.age_group == younger_label].iloc[0]
    o = g[g.age_group == older_label].iloc[0]
    # The grouped rows must first be collapsed within each age category.
    yg = g[g.age_group == younger_label]
    og = g[g.age_group == older_label]
    y_tested = int(yg.tested.sum())
    o_tested = int(og.tested.sum())
    y_pos = int(yg.hpv_positive.sum())
    o_pos = int(og.hpv_positive.sum())
    y_multi = int(yg.multiple_primary.sum())
    o_multi = int(og.multiple_primary.sum())
    pos_table_s = np.array(
        [[y_pos, y_tested - y_pos], [o_pos, o_tested - o_pos]], dtype=int
    )
    mult_table_s = np.array(
        [[y_multi, y_pos - y_multi], [o_multi, o_pos - o_multi]], dtype=int
    )
    pos_fisher_s = fisher_exact(pos_table_s)
    mult_fisher_s = fisher_exact(mult_table_s)
    stratified.append(
        {
            "cytology": cytology,
            "younger": {
                "tested": y_tested,
                "hpv_positive": y_pos,
                "multiple": y_multi,
            },
            "older": {
                "tested": o_tested,
                "hpv_positive": o_pos,
                "multiple": o_multi,
            },
            "hpv_positivity_older_vs_younger": {
                **odds_ratio_older_vs_younger(pos_table_s),
                "fisher_p": float(pos_fisher_s.pvalue),
            },
            "multiple_older_vs_younger": {
                **odds_ratio_older_vs_younger(mult_table_s),
                "fisher_p": float(mult_fisher_s.pvalue),
            },
        }
    )
result["cytology_stratified"] = stratified

# NILM women were tested electively during preventive examinations. This
# subgroup is analyzed as an opportunistic prevalence sample, not as a
# nationally representative probability sample.
nilm = df[df.cytology == "NILM"].copy()
nilm_summary_rows = []
for group, g in nilm.groupby("age_group", sort=False):
    tested = int(g.tested.sum())
    positive = int(g.hpv_positive.sum())
    multiple = int(g.multiple_primary.sum())
    pos_ci = wilson(positive, tested)
    mult_ci = wilson(multiple, positive)
    nilm_summary_rows.append({
        "age_group": group,
        "tested": tested,
        "hpv_positive": positive,
        "hpv_negative": tested - positive,
        "prevalence": positive / tested,
        "prevalence_ci_low": pos_ci[0],
        "prevalence_ci_high": pos_ci[1],
        "multiple": multiple,
        "single": positive - multiple,
        "multiple_fraction": multiple / positive,
        "multiple_ci_low": mult_ci[0],
```

```
        "multiple_ci_high": mult_ci[1],
    })
nilm_summary = pd.DataFrame(nilm_summary_rows)
ny = nilm_summary[nilm_summary.age_group == younger_label].iloc[0]
no = nilm_summary[nilm_summary.age_group == older_label].iloc[0]
nilm_pos_table = np.array(
    [[ny.hpv_positive, ny.hpv_negative], [no.hpv_positive, no.hpv_negative]],
    dtype=int,
)
nilm_mult_table = np.array(
    [[ny.multiple, ny.single], [no.multiple, no.single]], dtype=int
)
nilm_pos_chi = chi2_contingency(nilm_pos_table, correction=False)
nilm_pos_fisher = fisher_exact(nilm_pos_table)
nilm_mult_chi = chi2_contingency(nilm_mult_table, correction=False)
nilm_mult_fisher = fisher_exact(nilm_mult_table)

overall_nilm = df[df.cytology == "NILM"]
overall_tested = int(overall_nilm.tested.sum())
overall_positive = int(overall_nilm.hpv_positive.sum())
overall_ci = wilson(overall_positive, overall_tested)
result["nilm_preventive_subgroup"] = {
    "overall": {
        "tested": overall_tested,
        "hpv_positive": overall_positive,
        "prevalence": overall_positive / overall_tested,
        "ci_low": overall_ci[0],
        "ci_high": overall_ci[1],
    },
    "summary": nilm_summary.to_dict(orient="records"),
    "prevalence_comparison": {
        **odds_ratio_older_vs_younger(nilm_pos_table),
        "pearson_chi2": float(nilm_pos_chi.statistic),
        "pearson_p": float(nilm_pos_chi.pvalue),
        "fisher_p": float(nilm_pos_fisher.pvalue),
    },
    "multiple_comparison": {
        **odds_ratio_older_vs_younger(nilm_mult_table),
        "pearson_chi2": float(nilm_mult_chi.statistic),
        "pearson_p": float(nilm_mult_chi.pvalue),
        "fisher_p": float(nilm_mult_fisher.pvalue),
    },
}

OUT_JSON.write_text(json.dumps(result, indent=2), encoding="utf-8")
summary.to_csv(OUT_CSV, index=False)
print(json.dumps(result, indent=2))
```
